# The Therapeutic Function of Music Plan: a tool to specify the theory of music in music-based interventions

**DOI:** 10.3389/fpsyg.2025.1577176

**Published:** 2025-05-13

**Authors:** Deanna Hanson-Abromeit

**Affiliations:** Baby Music Intervention Research Lab, Department of Music Education and Music Therapy, School of Music, University of Kansas, Lawrence, KS, United States

**Keywords:** therapeutic function of music, theory of music, music-based intervention, intervention development, research

## 1 Introduction

“Why music?” is a question I regularly ponder. As a music therapist, educator and researcher I wonder *how* music creates change in people and *why* music matters. This curiosity drives my work and resulted in developing the Therapeutic Function of Music Plan (or TFM), an organizational matrix to justify and articulate the purpose and descriptive features of essential music elements within an intervention (Hanson-Abromeit, 2015). Originally designed to assist students and clinicians in articulating the role of music in music therapy, the purpose of this opinion article is to highlight the TFM and its potential to improve the rigor of MBI development, implementation and research.

Interest in music-based interventions (MBIs) has grown significantly in the last decade. In the United States, this growth was supported by the Sound Health initiative (https://www.nih.gov/research-training/medical-research-initiatives/sound-health), which led to targeted National Institutes of Health (NIH) funding for MBI research. There is a call to deepen the scientific rigor of music-based interventions to improve understanding of how and why music is effective in changing human behavior. The *NIH Music-based Intervention Toolkit* (Edwards et al., 2023) defines parameters for the development and evaluation of MBIs, building consistent research that moves science of MBIs forward. Notably, the toolkit advocates for identification of specific music elements and their incorporation into components of MBIs.

## 2 Function of music

The relationship between music and changes in human behavior beyond a casual understanding of music has long been known. Music therapists are responsive to the nuances of human behavior reflected through and with the music for therapeutic function. Early ethnomusicologist Merriam (1964) defined 10 functions of music in society: Emotional expression, aesthetic enjoyment, entertainment, fostering meaningful communication, symbolic representation, physical response, enforcing conformity to social norms, validating social institutions and stability of culture, contributing to both the continuity and stability of culture through shared values, and the integration of society through positive social behaviors. Music therapy authors built on Merriam's functions of music to guide therapeutic applications of music within clinical settings (Gfeller, 2008). There is continued interest in exploring functions of music for people within clinical settings.

For example, the Therapeutic Music Capacities Model articulates evidence-based research to support seven capacities of music for people with specific neurological disorders as engaging, emotional, physical, personal, social, persuasive, and facilitating synchronization (Brancatisano et al., 2020). For adolescents and young adults with autism spectrum disorder, music has cognitive, emotional, social and identity functions (Kirby and Burland, 2022). Moreover, retrospective approaches examine relationships in the music and behavior (e.g., Bruscia, 1987, 1988), and systems exist to select pre-recorded music for health outcomes (e.g., Rossetti, 2014) or create detailed descriptions of music therapy interventions (Hakvoort and Tönjes, 2023). Review studies look for patterns in how music functions for different populations (e.g., Lepping et al., 2024; Martin-Saavedra et al., 2018; Papatzikis et al., 2024; Robb et al., 2018). This research makes important contributions to understanding the function of music. However, despite the availability of guidelines for reporting music-based interventions (Robb et al., 2011), gaps exist in strong theories to support how music functions for therapeutic application. A bigger gap is our understanding and systematic application of the distinct features of the music for therapeutic outcomes. The TFM can potentially address existing gaps that limit MBI advancements.

Best practices in intervention development recommend connecting theories related to outcome, change, and intervention for stronger efficacy of interventions (Sidani and Braden, 2021). Integrated theories in intervention development include theory of health problem (articulates population need and factors that can be changed), theory of change (identifies the proposed mechanism), and the theory of intervention (defines intervention components) (Sidani and Braden, 2021). Within MBIs, adding theory of music communicates the relationship and levels of complexity of music elements (e.g., rhythm, tempo) to the health need, mechanism and intervention components. Distinguished from traditional music theory, which identifies common patterns and methods for “rules” of creating and performing music, theory of music hypothesizes how and why music actively contributes to change (Hanson-Abromeit, 2015; Hanson-Abromeit and Sena Moore, 2022). Thus, theory of music guides the musical features specific to MBIs outcome and mechanism.

## 3 Therapeutic Function of Music Plan

**Figure 1:**
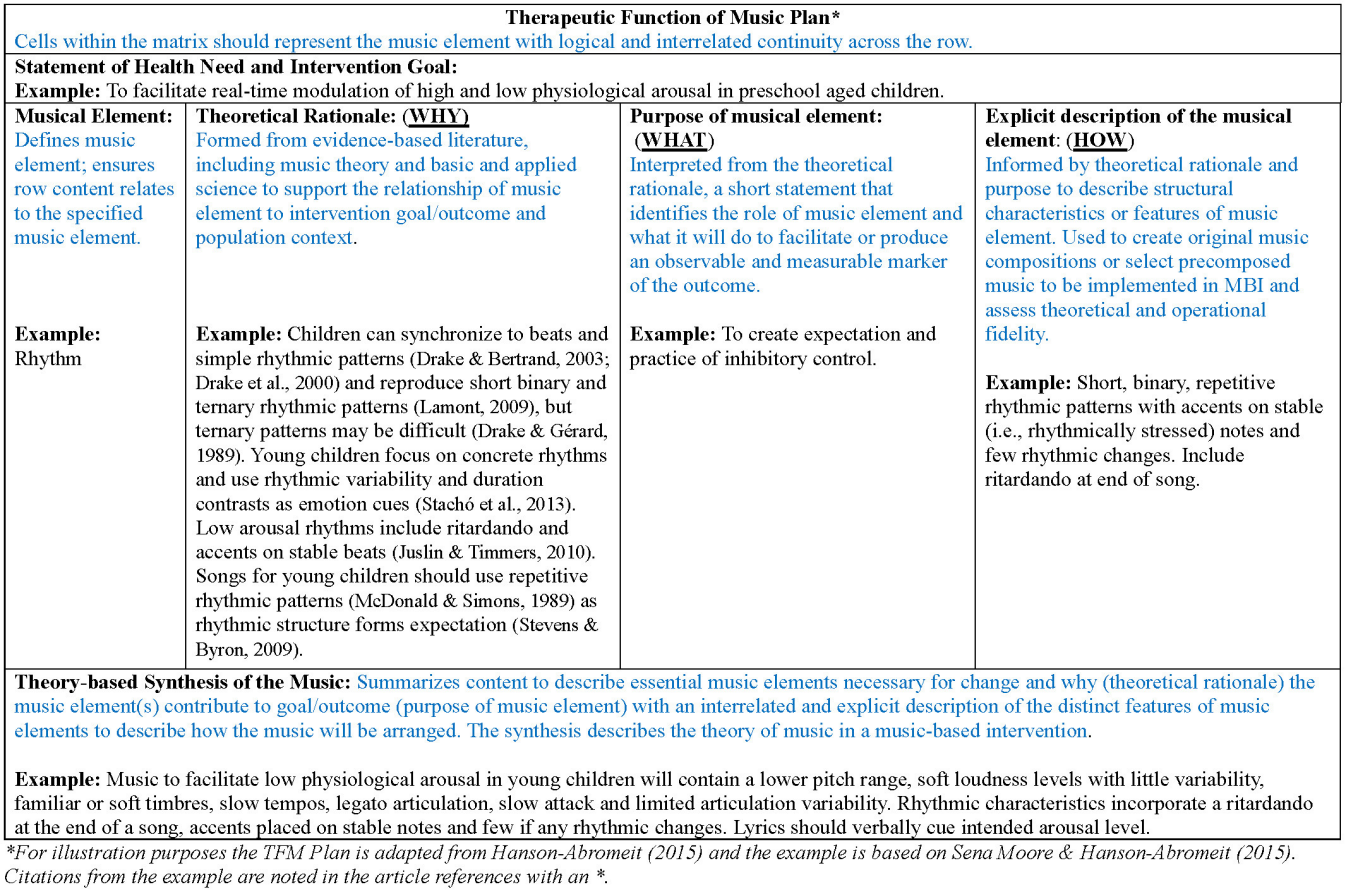
Annotated Therapeutic Function of Music Plan. For illustration purposes the TFM plan is adapted from Hanson-Abromeit (2015) and the example is from Sena Moore and Hanson-Abromeit (2015). Hanson-Abromeit, D. (2015). A conceptual methodology to define the Therapeutic Function of Music. *Music Therapy Perspectives*, 33(1), 25–38. doi: 10.1093/mtp/miu061, by permission of Oxford University Press and the American Music Therapy Association.

The TFM is an organizational matrix that connects a theoretical premise built from music theory, basic, and applied sciences to inform the purpose of core music elements deemed to target population specific outcomes. The TFM clarifies essential music elements and explicitly describes features of these music elements. A synthesis of this information summarizes the theory of music, which informs music selection or original musical composition and reporting details of MBIs (Hanson-Abromeit, 2015). Figure 1 illustrates the TFM matrix with a sample based on a published TFM (Sena Moore and Hanson-Abromeit, 2015) and construction details can be found in Hanson-Abromeit (2015). An example TFM and application to the development and evaluation of a novel MBI can be found in related publications (see Sena Moore and Hanson-Abromeit, 2015, 2018, 2023, 2024). Music attributes highlighted in the TFM supports rigor in MBI development and is an existing tool to advance research of MBIs.

## 4 Relating the TFM to the music-based intervention toolkit

The NIH MBI toolkit (Edwards et al., 2023) recommends seven components to advance rigor and scientific value of MBIs. These include a conceptual framework, clear research questions, supporting data to assess the hypotheses, common elements or building blocks of the MBI, a detailed description of the MBI, details of intervention delivery, information on the intervention population, and comparison or control groups. The TFM offers a solution to improving MBI development and evaluation aligned with the NIH MBI toolkit.

The TFM contributes to the conceptual framework of MBIs by specifying what the music contributes to the intended outcomes (i.e., theory of music) and is connected to the theory of problem, theory of change and theory of intervention for an identified population. The TFM defines the features of the music elements from supporting evidence within the theoretical rationale directly addressing the MBI toolkit recommendations to “focus on the components of the intervention…and in MBIs, this focus would include music elements…” (p. 871). Detailed description of the music and the purpose of the music elements defined by the TFM supports improved reporting details of MBIs and advances consistent replication and translation into efficacious clinical practice.

Moreover, the TFM offers deeper exploration of MBI delivery, including operational and theoretical fidelity. Theoretical fidelity assesses an intervention's essential elements, components, activities and actions through expert evaluation of content validity (i.e., how well the components address the factors identified for change) (Ibrahim and Sidani, 2016). In MBIs, theoretical fidelity should also assess the theoretical strength of the intervention's music. For example, the music selected for the intervention, either pre-composed or original musical compositions, can be evaluated for how well it follows the described features of the music within the TFM. These features form the essential music elements hypothesized to address the outcomes, and if present within the music should foster stronger outcomes. Theoretical fidelity leads to stronger consistency between theory, fidelity, and effective interventions. It can expand research centered on the music itself, ultimately improving both understanding of MBIs and implementation in clinical practice. To date, direct comparisons of music in MBIs with and without theoretical fidelity have not been explored.

Theoretical fidelity of the MBI music strengthens criteria for implementation fidelity, a need in MBIs (Wiens and Gordon, 2018). The TFM explicit descriptions of the music elements provide observable and measurable features of the music so the facilitation of the music can be measured through implementation fidelity. Implementation fidelity examines whether the interventionist provided components of the intervention but rarely examines consistency in the presentation of the music characteristics during implementation of the intervention. The TFM specifies the characteristics of the music elements that can be used to identify the musicianship necessary for implementation of the music by the interventionist. For example, in the Musical Contour Regulation Facilitation (MCRF) MBI (Sena Moore and Hanson-Abromeit, 2018), some of the songs require complex guitar accompaniment patterns at faster tempos, thus demanding higher skills in guitar accompaniment by the interventionist. Moreover, detailed characteristics of the music elements in the TFM creates observable features of the music so that consistent implementation can be assessed and measured. Such detail in describing the qualities of the music elements and implementation factors can further support the identification and reporting of interventionist qualifications differentiating between MBIs that require a music therapist and those that could be implemented by another specialist or self-administered. Examining the congruency of the music-based implementation fidelity across studies and interventionists may lead to greater information on the efficacy of the intervention and therapist effectiveness, thus strengthening the details and essential characteristics of the MBI for the effectiveness.

The relationship of the music and participant responses is also an important research question to strengthen the detail of the protocol and intervention manual. In MBIs there is an assumption that music contributes to change. When we evaluate outcomes of participant behaviors in MBIs (e.g., Sena Moore and Hanson-Abromeit, 2023), we also want to evaluate how responsive participants are to the intervention, including the music. The TFM tightly connects the variables of behavior change expected in participants to the variables of the music. This could help strengthen the theoretical premise of the intervention identified through the interconnected theories of health problem, mechanism, music and intervention and provide insight into optimization of an intervention without losing its essence and effectiveness.

The TFM could also guide active controls in clinical trials. The TFM specifies the features of the music elements hypothesized to be essential for outcomes. Strong theoretical fidelity ensures these features are evident in the MBI's musical compositions. Removing TFM specified features from the music used in active control conditions distinguishes the music in the MBI from the control while maintaining music in both conditions. This distinction could deepen understanding of how music contributes to change, move researchers beyond a generalized description and application of music in MBIs, and lead to improved rigor in randomized control trials. Furthermore, improved control group comparisons, could further support the translation and wider acceptability of music-based interventions as an essential therapeutic modality.

## 5 Discussion

The Therapeutic Function of Music Plan (TFM) is not the first attempt to identify the relationship between music characteristics and therapeutic outcomes. However, it is unique in hypothesizing the theory of music. Built from interdisciplinary applied and basic sciences, the TFM defines the theoretical relationship between music elements, illustrating how and why music functions as the primary modality in the intervention and activates the hypothesized or known mechanism of change. The TFM informs the components and essential music elements of MBIs at the intervention development phase. Thus, the TFM adds to current knowledge and contributes to the interconnectedness of MBI design and development.

Initially developed as a teaching and clinical tool to build knowledge for clinical decision making, the TFM was published in a clinical journal a decade ago (Hanson-Abromeit, 2015). Explorations of the TFM in clinical practice and training are emerging in US-based music therapy programs. Currently, there are no published studies examining differences in MBIs with and without the TFM. Its application in research is in the early stages (e.g., Fiore, 2018; Reschke-Hernández et al., 2023; Sena Moore and Hanson-Abromeit, 2015, 2018, 2023, 2024; Sena Moore et al., 2025). The purpose of this opinion article is to raise awareness of the TFM's potentiality and encourage others to explore its functionality.

As a matrix, the TFM has flexibility for different philosophical approaches, emerging conceptual and theoretical rationale, populations, and outcomes. It requires deeper questions about the therapeutic function of music and subcomponent parts (i.e., rhythm, tempo, pitch, etc.). Strengthening theory in MBIs will create greater specificity in the music within MBIs, proactively address existing gaps, and strengthen scientific rigor. The TFM fills a gap clearly articulated as important to increasing the science of MBIs. The TFM is an existing tool useful for anyone conducting music-based intervention development and research and contributes to advancing discourse, rigor and efficiency in research and development of future MBIs.
